# Regulatory role of RNA N^6^-methyladenosine modifications during skeletal muscle development

**DOI:** 10.3389/fcell.2022.929183

**Published:** 2022-08-05

**Authors:** Baojun Yu, Jiamin Liu, Juan Zhang, Tong Mu, Xiaofang Feng, Ruoshuang Ma, Yaling Gu

**Affiliations:** College of Agriculture, Ningxia University, Yinchuan, China

**Keywords:** N6-methyladenosine (m^6^A) modification, myogenesis, skeletal muscle development, transcriptional regulation, epigenetic

## Abstract

Functional cells in embryonic myogenesis and postnatal muscle development undergo multiple stages of proliferation and differentiation, which are strict procedural regulation processes. N^6^-methyladenosine (m^6^A) is the most abundant RNA modification that regulates gene expression in specific cell types in eukaryotes and regulates various biological activities, such as RNA processing and metabolism. Recent studies have shown that m^6^A modification-mediated transcriptional and post-transcriptional regulation plays an essential role in myogenesis. This review outlines embryonic and postnatal myogenic differentiation and summarizes the important roles played by functional cells in each developmental period. Furthermore, the key roles of m^6^A modifications and their regulators in myogenesis were highlighted, and the synergistic regulation of m^6^A modifications with myogenic transcription factors was emphasized to characterize the cascade of transcriptional and post-transcriptional regulation during myogenesis. This review also discusses the crosstalk between m^6^A modifications and non-coding RNAs, proposing a novel mechanism for post-transcriptional regulation during skeletal muscle development. In summary, the transcriptional and post-transcriptional regulatory mechanisms mediated by m^6^A and their regulators may help develop new strategies to maintain muscle homeostasis, which are expected to become targets for animal muscle-specific trait breeding and treatment of muscle metabolic diseases.

## 1 Introduction

The skeletal muscle is mainly composed of a large number of muscle fibers, a small amount of adipose tissue, and connective tissue, which are highly heterogeneous striated muscles that contain muscle cells, immune cells, and nerves. Accounting for approximately 40% of an animal’s body weight, the skeletal muscle is the organism’s largest motor and metabolic organ and plays a vital role in metabolism and energy balance (Mayeuf-Louchart et al., 2015; Cong et al., 2020). Embryonic myogenesis and postnatal muscle development are precisely regulated by multiple mechanisms that involve myoblast progenitor cell proliferation, differentiation, and fusion to form muscle fibers (Zhu et al., 2021). Skeletal muscle development involves multiple stages of proliferation and differentiation, and research on its formation process and molecular regulation mechanisms has always been a hot topic in the field of molecular genetics. Currently, research on skeletal muscle growth and development mainly focuses on the functional identification of key genes and the post-transcriptional regulatory mechanisms mediated by non-coding RNAs (such as lncRNA, circRNA, and microRNA) (Zhao et al., 2011). In addition to being regulated by a series of specific transcription factors and signaling pathways, epigenetic modifications are also involved in a variety of biological processes in muscle development (McKinnell et al., 2008; Yang et al., 2021). As the most common methylation modification in RNA (Deng et al., 2018), N^6^-methyladenosine (m^6^A) represents a new type of post-transcriptional gene regulation, which is tissue specific and spatio-temporally specific.

To date, more than 150 different RNA modifications have been identified (Yang et al., 2018), which play an important role in tissue development and homeostasis by controlling the cell state transition (Frye et al., 2018). The m^6^A modification is the most abundant methylation modification in eukaryotic cells, and it is widely distributed in mRNA and non-coding RNA and can regulate gene expression through “epitranscriptomics” without changing the sequence of RNA molecules (Roundtree et al., 2017a; Helm and Motorin, 2017; Fazi and Fatica, 2019). m^6^A modifications in eukaryotic RNA are reversible (Jia et al., 2012) and can be deposited, removed, and recognized by a series of methyltransferase complexes (writers), demethylases (erasers), and m^6^A-binding proteins (readers) (Lu et al., 2019). They are involved in the regulation of biological processes, such as disease occurrence (Han et al., 2019; Wang et al., 2020), embryonic development (Mendel et al., 2018), tissue development, and cell proliferation and differentiation (Zhang et al., 2017; Lee et al., 2019) by regulating RNA metabolic activities such as precursor RNA splicing (Haussmann et al., 2016), mRNA translocation (Fustin et al., 2013), stability (Geula et al., 2015), and translation (Coots et al., 2017). However, the post-transcriptional mechanisms of m^6^A modifications in the regulation of muscle development remain largely unknown.

Recent studies have shown that m^6^A methylation plays an important role in muscle stem cell maintenance, myocyte proliferation, and cell differentiation (Kudou et al., 2017; Wang et al., 2017; Dorn et al., 2019; Mathiyalagan et al., 2019; Gheller et al., 2020; Lin et al., 2020; Zhang et al., 2020). This review aims to provide an overview of the mechanisms of myogenesis under transcriptional and m^6^A methylation-mediated post-transcriptional regulation and emphasizes the important role of m^6^A modification and its regulators in coordinating functional signaling factors at different stages of skeletal muscle development. Based on the latest research in epigenetic modifications, this study also discusses the interaction between m^6^A modification and non-coding RNA and explores different levels of co-regulation in myogenesis.

## 2 Biological characteristics of skeletal muscle development

Both embryonic myogenesis and postnatal muscle development undergo a series of cell proliferation and differentiation processes from the embryonic stage to early growth and development, and mesoderm cells undergo repeated mitosis and massive proliferation to form mononuclear myoblasts. Furthermore, myoblasts proliferate and fuse into multinucleated myotubes (Velleman et al., 2013). During myogenesis, mononuclear myoblasts withdraw from the cell cycle, lose their ability to divide, and migrate from the cell center to the cell membrane to form myofibers (Picard et al., 2010). Single myoblasts that cannot be fused are isolated between the myofiber basement membrane and muscle cell membrane and finally form muscle satellite cells (MuSCs) with stem cell characteristics. The proliferative capacity of MuSCs decreases with age during animal growth and development. However, MuSCs can proliferate and undergo myogenic differentiation when myofibers are damaged, participating in their repair and regeneration (Zhang et al., 2018), thus ensuring normal growth and development of animals.

### 2.1 Embryonic myogenesis

**FIGURE 1:**
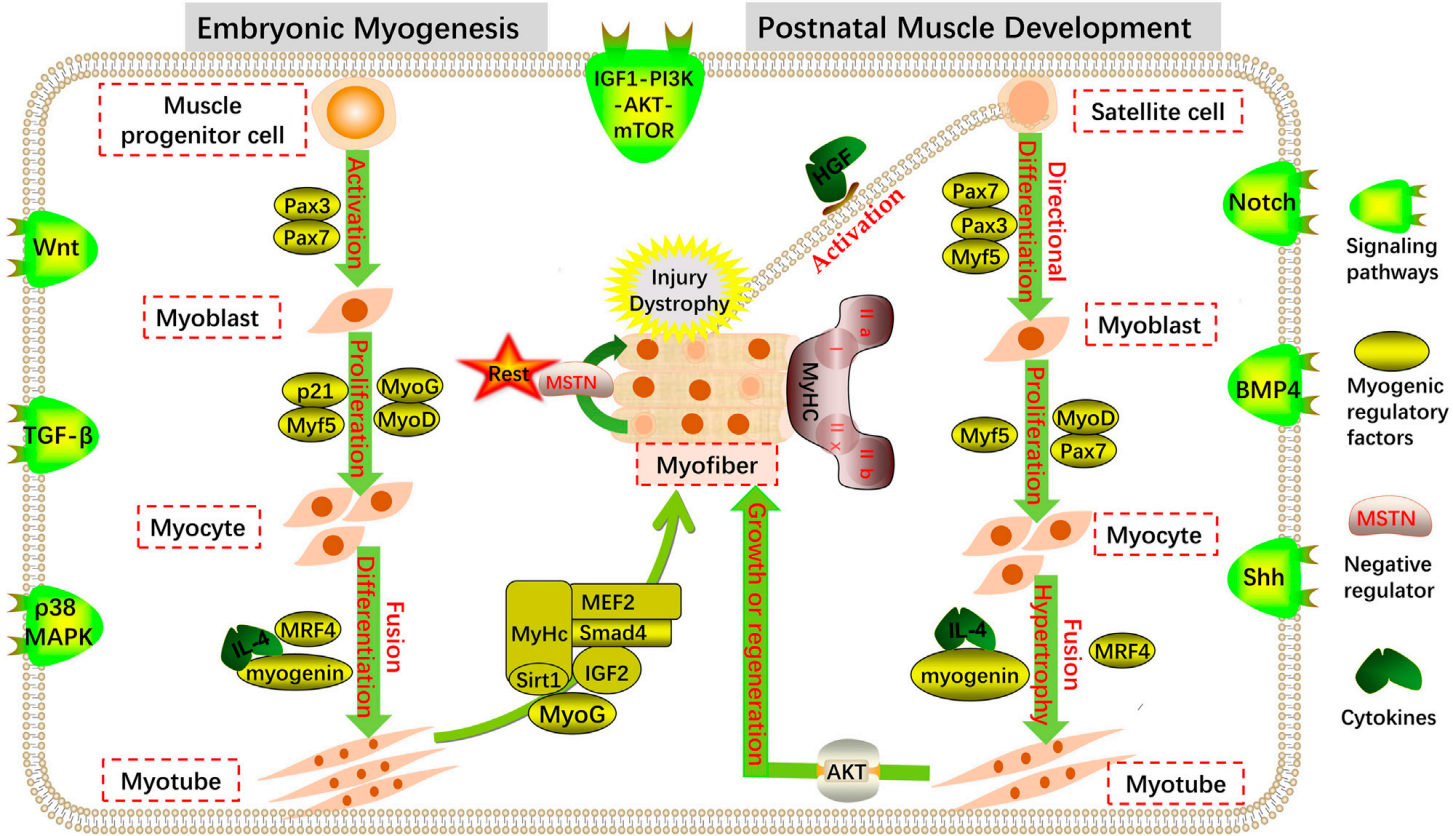
Regulation of myogenesis. Coordinated expression of multiple transcription factors and signaling pathways at different stages of embryonic myogenesis and postnatal muscle development maintain the dynamic balance of muscle cell proliferation and differentiation.

The genesis and development of the skeletal muscle in the animal embryo is a complex physiological and biochemical process that primarily includes muscle cell genesis, myofiber formation and maturation, and the accumulation of myofiber number (Figure 1). The skeletal muscles of vertebrates originate from the paraxial mesoderm during embryonic development (Buckingham and Vincent, 2009). The paraxial mesoderm differentiates to produce somites, which act as the “helmsman of fate” and control the direction of myogenesis and osteogenesis (Biressi et al., 2007). The somite matures and divides again during embryo development. Driven by the sonic hedgehog (Shh) signal secreted by the neural tube and notochord, a part of the epithelial cells of the dorsal develops into a dermomyotome (Buckingham, 2001). Pluripotent stem cells located in the somite differentiate into muscle progenitor cells (MPCs) under the conditions of various signal molecules and embryonic environments. With the proliferation of dermomyotome cells, the number of MPC increases continuously, and the MPC in the middle of the dermomyotome continues to migrate downward to form the first skeletal muscle tissue: the myotome. MPCs are further exfoliated from the dermomyotome and fused with the myotome to form skeletal muscle, and some MPCs migrate to the extremities to form the limb skeletal muscle (Parker et al., 2003; Jensen et al., 2010).

During primary muscle development, MPC-expressing Pax3, Pax7, and low Myf5 genes in the myotome stratify from somites and migrate to more distant muscle tissues to form mononuclear myoblast precursors, which are then induced by myogenic determinants to differentiate further into myoblasts (Kassar-Duchossoy et al., 2005; Bajard et al., 2006). During the development of the secondary muscle in the later stage of the embryo, myoblasts proliferate, and the expression of the muscle differentiation factor, myogenin, and other genes increases (Tajbakhsh and Buckingham, 2000). After a series of proliferation, myoblasts withdraw from the proliferation cycle and are irreversible. Then, they undergo terminal differentiation by expressing muscle-specific proteins and different types of cell adhesion factors and fuse to form fusiform multinucleated myotubes containing non-striated myofibrils (Walsh and Perlman, 1997; Sabourin and Rudnicki, 2000). The multinucleated cells already contain myofibrils composed of myosin and actin. When myofibril filaments are arranged in rows to produce transverse striations, the myotubes further develop into mature myofiber-forming skeletal muscles with a relatively perfect structure and function (Buckingham et al., 2003; Buckingham, 2006; Buckingham and Relaix, 2007). The embryonic stage is the main period of myofiber quantity fixation and differentiation in most mammals.

### 2.2 Postnatal skeletal muscle development

The number of skeletal myofibers after birth is fixed, and muscle growth is mainly derived from increased cell volume (Sassoon, 1993). In this process, the length of myofibers increases with the length and number of sarcomeres, and at the same time, myofibrils increase the diameter of myofibers through multiple divisions. Notably, MuSCs are essential for the developmental growth of myofibers, which can replenish the nuclei of the postnatal myocyte pool, thus contributing to the increase in myonuclei during the early postnatal stage (Pallafacchina et al., 2013; Fukada and Ito, 2021). When some MuSCs divide and proliferate, their nuclei fuse with myofibers, maintaining the relative balance between myocyte nuclei and cytoplasm and promoting myocyte enlargement, thus causing the skeletal muscle to exhibit a growth state (Pallafacchina et al., 2013; Fukada and Ito, 2021). MuSCs are pre-eminent stem cells that maintain the regeneration of postnatal myofibers and are generally in a quiescent state of mitosis (Partridge, 2004; Dhawan and Rando, 2005). However, when mature myofibers are damaged, MuSCs can be activated and re-enter the myogenic pathway, differentiate into myoblasts, and fuse to produce new myofibers after a series of proliferation and differentiation processes (Collins et al., 2005; Dhawan and Rando, 2005; Le Grand and Rudnicki, 2007; Tedesco et al., 2010) (Figure 1).

The growth and development of the postnatal skeletal muscle are accompanied by the transformation and maturation of myofiber types. According to the different expression activities of ATP enzymes in myofibers, myofibers are divided into slow oxidation, fast oxidation, fast glycolysis, and intermediate oxidation types (Schiaffino and Reggiani, 1996). It has been discovered that in slow myofiber activity, unlike in fast myofibers, protein synthesis and degradation rates are higher (Li and Goldberg, 1976). Furthermore, muscle activity is mainly reflected in protein metabolism, and the regulation of muscle mass and myofiber size mainly depends on the balance between protein synthesis and degradation in myofibers (Goll et al., 2008; Yin et al., 2021). When myofibers are stimulated by load or synthetic metabolic hormones after development and maturity, the total protein synthesis rate of the skeletal muscle is greater than the degradation rate, and the size of myofibers increases to promote muscle growth. When an organism is starved, diseased, or stimulated by catabolic hormones, the synthesis rate of skeletal muscle protein is reduced, resulting in the loss of the balanced state of synthesis and degradation and therefore decreased skeletal muscle mass and muscle atrophy (Burd et al., 2010; Goodman, 2014; Mckendry et al., 2021; Sartori et al., 2021).

### 2.3 The critical role played by muscle cells

Totipotent muscle cells (myocytes) exist throughout skeletal muscle growth and development during the embryonic and postnatal stages of vertebrates, and the myocytes in the somites begin to be active in the early stages of myogenesis. During various stages of animal life, myocytes promote the formation and development of muscle tissue through a series of proliferation and differentiation processes (Figure 1).

#### 2.3.1 Myoblasts

Myoblasts are derived from muscle progenitor cells, which are the basic materials for myofiber formation. The proliferation and differentiation of myoblasts play an important role in muscle development. After muscle injury, stationary MuSCs activate and commit to myoblasts and further proliferate, fuse, and eventually mature into myofibers and restore the contraction ability of injured muscles (Zhang et al., 2018). Therefore, myoblasts are the driving force of skeletal muscle development and an effective tool for treating many diseases with poor prognoses, such as clinical muscular atrophy.

During secondary muscle development, myoblast proliferation and fusion are regulated by a variety of molecular networks. Myoblasts play a decisive role in muscle development under the direct or indirect action of a series of regulatory factors. Owing to the fundamental constitutive role of myoblasts in muscle development, maintenance, and adaptation, the study of regulatory mechanisms of myoblast differentiation has become a key direction in skeletal muscle growth and development, and as an *in vitro* model, it is widely used in muscle growth, differentiation, migration, apoptosis, and other related studies (Sharples and Stewart, 2011; Cai et al., 2020).

#### 2.3.2 Satellite cells

MuSCs are myoblast precursor cells with the ability to proliferate and self-renew in the later stages of embryonic development and play an essential role in the growth and regeneration of newborn muscle (Chargé and Rudnicki, 2004; Endo, 2007; Relaix and Zammit, 2012; Yin et al., 2013). MuSCs are in the G0 phase and typically remain quiescent. When the skeletal muscle is stimulated externally, the basement membrane secretes the hepatocyte growth factor (HGF), which binds to receptors on the surface of MuSCs and activates quiescent-stage MuSCs (Tatsumi et al., 1998). Furthermore, under the induction of HGF and other related factors, some activated MuSCs re-enter the cell cycle and migrate along the myofiber to the damaged site (Bischoff, 1997). MuSCs proliferate massively after migrating to the injury site and generate sufficient myoblasts, which subsequently proliferate and fuse under the regulation of a series of muscle-specific factors to further develop into mature myofibers for skeletal muscle growth, maintenance, and regeneration (Halevy et al., 2004; Zammit et al., 2004; Rizzi et al., 2012). Inactive MuSCs re-enter the quiescent state and become reserve cells for the next cell cycle (Zammit et al., 2004). The self-renewal and myogenic differentiation of MuSCs maintain a dynamic balance during muscle development, which is essential for the normal function of MuSCs and the maintenance of homeostasis in the internal environment (De Luca et al., 2013).

In summary, the ability of MuSCs to remain quiescent plays a crucial role in the long-term maintenance of a functional stem cell pool during skeletal muscle development and regeneration. Myoblasts and MuSCs are the most important functional cells involved in muscle growth and development. The dynamic balance between their proliferation and differentiation is critical for maintaining the fate of skeletal muscle cells, thus ensuring normal growth and development of the organism.

## 3 RNA m^6^A-modified enzyme system

m^6^A is the most characteristic methylation modification in eukaryotic RNA, in which three key proteins—writers, erasers, and readers—are involved in maintaining the dynamic balance of its modification.

### 3.1 m^6^A methyltransferase (writers)

m^6^A methyltransferase is composed of the m^6^A–METTL core complex (MAC) and m^6^A–METTL-associated complex (MACOM) (Lence et al., 2019), and multiple subunits of the complex are co-transcribed and bound to RNA to catalyze methylation. The m^6^A–METTL complex includes methyltransferase 3 (METTL3) and methyltransferase 14 (METTL14), which can form stable heterodimers *in vitro*. METTL3 is highly conserved, including the SAM-binding domain and methyltransferase active domain, which catalyzes the formation of m^6^A (Wang et al., 2016a). METTL14 lacks the catalytic active domain of the enzyme but can promote the binding of METTL3 and RNA. When MELLT14 and METTL3 bind, the methylation catalytic ability of the complex is significantly enhanced (Wang et al., 2016b).

Further studies have shown that some m^6^A–METTL-associated complexes play an important role in guiding the methylation of specific target sites. Wilms’ tumor 1-associated protein (WTAP) lacks methylase activity, but it can promote m^6^A deposition by recruiting the METTL3–METTL14 complex to localize in nuclear plaques (Ping et al., 2014). Vir-like m^6^A methyltransferase associated (VIRMA) can promote the specific deposition of m^6^A in the 3′UTR (Yue et al., 2018). KIAA1429 also has the catalytic activity of m^6^A methylation, which affects splicing by regulating the level of m^6^A (Schwartz et al., 2014). RNA-binding protein 15 (RBM15) and its analog RBM15B contain three RNA recognition motif (RRM) domains that interact with the WTAP–METTL3 complex at specific sites to promote m^6^A methylation (Yang et al., 2018). CCCH type 13 zinc finger protein (ZC3H13) regulates the nuclear localization of the WTAP–Virilizer–Hakai complex and the self-renewal of mouse embryonic stem cells (ESCs) by promoting m^6^A methylation (Wen et al., 2018). Although the m^6^A modification mechanism of the key complex has been defined, future studies may identify additional subunits of the methyltransferase complex that promote or inhibit the occurrence of m^6^A by identifying specific loci and thereby accurately regulating gene expression.

### 3.2 m^6^A demethylase (erasers)

The m^6^A modification is a dynamic and reversible regulatory process whose activity can be counteracted by demethylases, fat mass and obesity-associated (FTO) gene, and ALKBH5 in gene regulation. The FTO gene is the first protease discovered to have m^6^A demethylation activity and belongs to the Fe (II) and α-KG (ketoglutaric acid)-dependent ALKB dioxygenase family. FTO exhibits catalytic activity both *in vitro* and *in vivo*. During demethylation, m^6^A is catalyzed to form N^6^-hydroxymethyladenosine (hm^6^A) and N^6^-formyladenosine (fm^6^A), which are extremely unstable and eventually decompose into adenine (A) (Jia et al., 2012; Fu et al., 2013). In the intracranial glioma model of nude mice, mice bearing FTO shRNA-1-infected U251 cells had significantly shorter survival times, and when mice were co-infected with FTO-Mut, they survived longer. This suggests that FTO inhibits the *in vivo* progression of gliomas (Tao et al., 2020). An ovariectomized mouse model demonstrated that FTO can promote osteoporosis by demethylating the osteogenic marker, Runx2 mRNA (Wang et al., 2021). In C57BL/6N mice, FTO deficiency results in weight loss, marked reduction in white adipose tissue, and promotion of the conversion of white adipocytes to brown or beige adipocytes (Ronkainen et al., 2015). Moreover, FTO-mediated mRNA m^6^A demethylation can affect preadipocyte differentiation and lipid deposition by regulating the expression of fat-related genes, such as C/EBPβ, PPARγ, and ANGPTL4, and plays an important regulatory role in lipid metabolism and lipid disorders (Yang et al., 2022b). It has been shown that inhibiting the expression of FTO can considerably increase the total m^6^A level of polyadenylated RNA (Jia et al., 2012). Additionally, FTO also uses N^6^,2′-O-dimethyladenosine (m^6^Am) on single-stranded RNA as a substrate and has higher demethylase activity (Mauer et al., 2017). ALKBH5, also derived from the ALKB protein family, is the second m^6^A demethylase discovered, and its catalytic activity is similar to that of FTO; however, it can directly demethylate m^6^A to A through one reaction. ALKBH5 plays a role in specific sequences, showing a preference for m^6^A in common sequences and can completely remove m^6^A methylation modifications from single-stranded RNA (Zheng et al., 2013).

### 3.3 m^6^A-binding protein (readers)

Following m^6^A modification in eukaryotes, respective downstream biological functions require specific recognition by reader proteins to proceed normally. Currently known m^6^A-binding proteins include the YT521-B homology (YTH) family, IGF2BP, and HNRNP.

The YTH domain family members mainly include YTHDF1/2/3 and YTHDC1/2, which contain a YTH domain that can selectively bind to the m^6^A site on RNA (Theler et al., 2014; Hsu et al., 2017). YTHDF2 has a strong binding ability, which can identify m^6^A sites and regulate the degradation of modified genes (Wang et al., 2014a). Studies have shown that YTHDF2 can enter the nucleus during heat stress stimulation, prevent FTO from de-modifying m^6^A in the 5′UTR, and promote translation in a non-hat-dependent manner (Zhou et al., 2015). YTHDF1 can interact with the translation initiation factor, eIF, to enhance the translation efficiency of m^6^A modified genes by promoting ribosome enrichment of methylated genes (Wang et al., 2015). YTHDF3 is the first reader protein that binds to nuclear-exported m^6^A-modified RNA, assisting YTHDF1 and YTHDF2 in regulating the degradation or translation of target genes, respectively (Li et al., 2017; Shi et al., 2017). However, recent studies have found that YTHDF1, YTHDF2, and YTHDF3 directly affect mRNA degradation in an m^6^A-dependent manner but do not participate in the translation regulation of mRNA (Lasman et al., 2020).

YTHDC1, located in the nucleus, is highly conserved, which can selectively recruit the pre-mRNA splicing factor, SRSF3, and promote its binding to m^6^A-modified mRNA. Furthermore, it inhibits the binding of SRSF10 and mRNA, promotes the retention of exons modified by m^6^A, and regulates mRNA splicing (Xiao et al., 2016). Additionally, YTHDC1 interacts with SRSF3 and nuclear RNA output factor 1 to promote the nuclear output of m^6^A-modified mRNA, which plays an important role in the metabolic regulation of mRNA (Roundtree et al., 2017b). YTHDC2 preferentially binds to m^6^A in a common motif to improve translation efficiency and reduce the abundance of its target mRNA (Yang et al., 2018). The insulin-like growth factor-2 mRNA-binding protein (IGF2BP) family consists of three homologous coding genes, IGF2BP1, IGF2BP2, and IGF2BP3, which contain an RNA recognition domain and a ribonucleoprotein K domain. IGF2BPs promote the stability and storage of target genes in an m^6^A-dependent manner through the ribonucleoprotein K domain (Huang et al., 2018). Finally, a family of HNRNP proteins mediates the “m^6^A-switch” mechanism, and its member, HNRNPA2B1, can directly bind to m^6^A and cooperate with METTL3 to regulate alternative splicing events and primary microRNA processing. The other two members, HNRNPC11 and HNRNPG, do not directly bind to m^6^A but regulate the processing of RNA transcripts containing m^6^A (Yang et al., 2018).

## 4 Regulation of skeletal muscle growth and development by RNA m^6^A modifications

**FIGURE 2:**
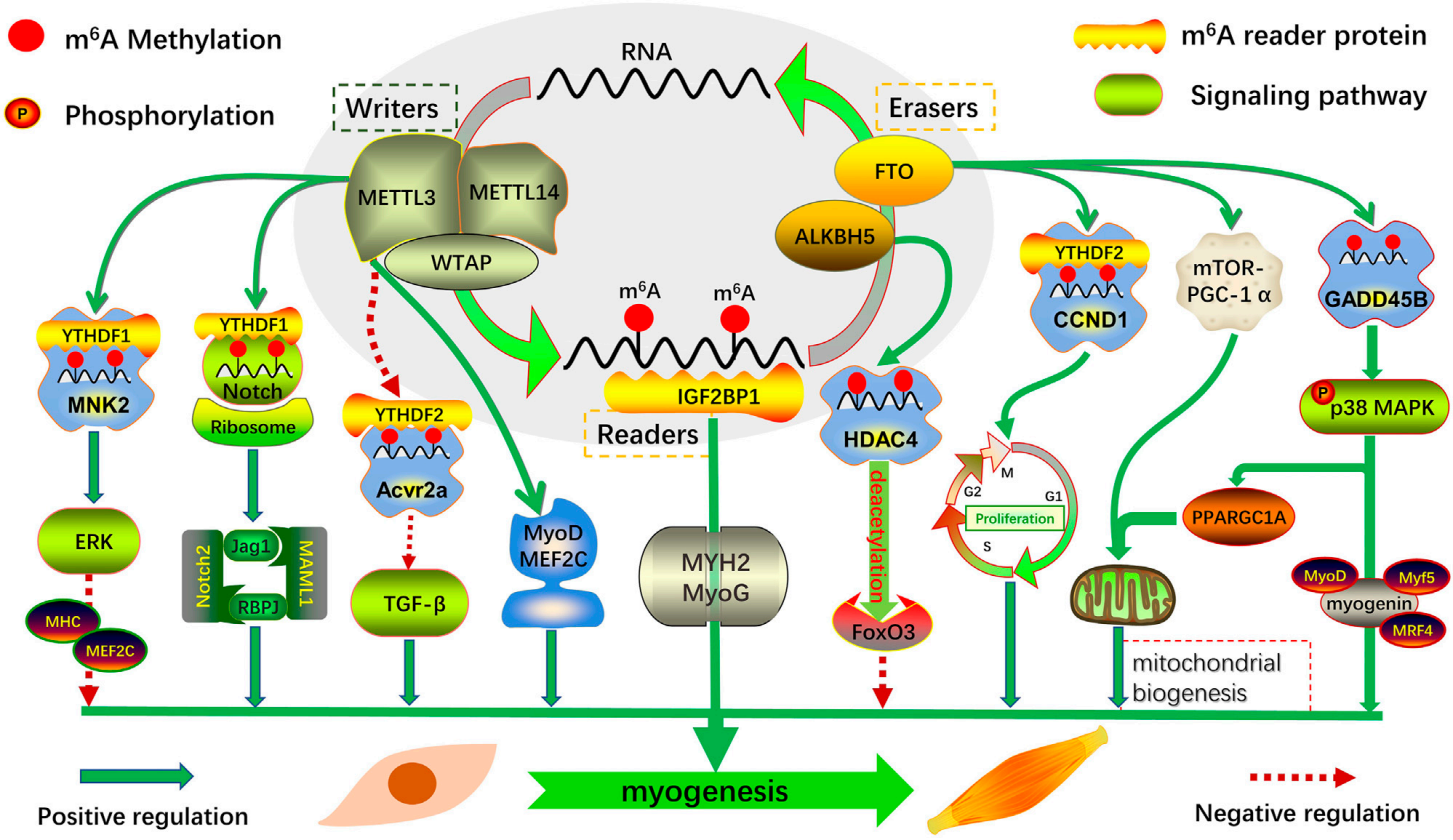
Regulatory role of m^6^A modification and its regulators in skeletal muscle myogenesis. Myogenic genes that mediate myogenesis are regulated transcriptionally and post-transcriptionally by m^6^A-related proteins in multiple ways: regulating the transcription of key factors in an m^6^A-dependent manner; directly regulating the expression of related transcription factors; and coordinating with other transcription factors or post-transcriptional modifications.

m^6^A modification regulates various biological activities, such as RNA processing and metabolism in eukaryotes. Its dynamic and reversible mode of action may affect gene expression and cell fate by regulating various RNA-related cellular signaling pathways. Given the role of m^6^A modification in gene expression and the regulation of functional cellular mechanisms, there is emerging evidence that m^6^A modification and its regulators play an essential role in the growth and development of the skeletal muscle (Kudou et al., 2017; Wang et al., 2017; Gheller et al., 2020; Zhang et al., 2020; Deng et al., 2021a; Li et al., 2021; Petrosino et al., 2022) (Figure 2). Therefore, this review focused on the cascade regulation of m^6^A modification during embryonic and postnatal skeletal muscle development to provide evidence for exploring a new mechanism of m^6^A regulation of myogenesis.

### 4.1 Embryonic m^6^A methylation

In recent years, many studies have shown that m^6^A plays an important role in regulating the embryonic development of eukaryotes. During early embryonic development in zebrafish, m^6^A modification promotes maternal–zygote transition through YTHDF2-dependent maternal mRNA clearance (Zhao et al., 2017). In addition, m^6^A participates in the metabolism of mRNA in embryonic stem cells, maintains cell self-renewal, and regulates the state transition and pluripotency of embryonic cells as a determining factor of cell fate at the transcriptional level (Batista et al., 2014).

The regulatory mechanism of m^6^A in embryonic cell fate, which exists in a tissue-specific form at different stages of embryonic development, has been continuously explored. It maintains the dynamic balance between biological processes by regulating functional gene transcription and post-transcriptional expression. Determining the number of skeletal muscle fibers in animal embryos involves complex regulatory mechanisms, and the expression of key muscle-specific transcription factors is precisely regulated by m^6^A methylation (Figure 2). Therefore, in-depth exploration of the potential mechanism of m^6^A in embryonic skeletal muscle development is of key interest in developmental muscle biology.

Analysis of two key stages of pectoral muscle development in Dingan goose embryos revealed a negative correlation between m^6^A methylation and gene expression. Moreover, most m^6^A-modified differentially methylated genes were significantly enriched in muscle-related pathways, such as the Wnt, mTOR, and FoxO signaling pathways (Xu et al., 2021). Combined with miRNA-seq, potential m^6^A-miRNA-PDK3 was screened, which revealed the key role of m^6^A-modified miRNA in muscle growth and development of Dingan goose embryos (Xu et al., 2021). Analysis of m^6^A distribution in goat muscles at two key developmental stages revealed that the m^6^A peak in the longissimus at embryonic day 75 was significantly higher than that in the newborn stage, and m^6^A-modified genes were mainly enriched in actin binding, myotubular differentiation, MAPK, Wnt, and other signaling pathways related to skeletal muscle development (Deng et al., 2021a). During the differentiation of goat primary myoblasts, FTO expression was negatively correlated with global m^6^A levels. Following FTO knockdown in myoblasts, m^6^A levels of GADD45B mRNA were increased, whereas its protein expression and phosphorylation levels of p38 MAPK were significantly decreased, and myotube formation was attenuated. This demonstrated that the FTO-mediated m^6^A demethylates GADD45B and activates the p38 MAPK pathway, which in turn promotes myogenic differentiation of goat skeletal muscle (Deng et al., 2021a).

The expression of IGF2BP1 is continuously downregulated in the six stages of skeletal muscle growth and development in the embryonic pig stage. Combined with RIP-seq, m^6^A-modified myogenic marker genes, MYH2 and MyoG, were identified as target genes of IGF2BP1 (Zhang et al., 2020). Loss-of-function experiments were performed in myoblasts, and it was found that knocking down IGF2BP1 significantly downregulated MYH2 and MyoG mRNA expression and significantly inhibited myotube formation. The same phenotypic changes were observed with METTL14 knockdown (Zhang et al., 2020), demonstrating that m^6^A is a key epigenetic factor in embryonic myogenesis. These results suggest that dynamic changes in m^6^A modification levels during embryonic skeletal muscle development play an essential role in regulating myogenesis.

### 4.2 Postnatal m^6^A methylation

Studies have found that m^6^A methylation has commonality and discrepancy in regulatory functions in different developmental stages of the organism, and it also plays an essential role in postnatal skeletal muscle development and muscle regeneration (Figure 2). Through whole-transcriptome m^6^A methylation map analysis of the muscle tissue of wild boar and Landrace and Rongchang pigs, it was found that most of the nuclear-related genes containing m^6^A encode transcription factors, indicating that m^6^A modification is involved in transcriptional regulation, and the two coordinately regulate gene expression (Tao et al., 2017). This is consistent with the results of a recent study on m^6^A profiles in the longissimus dorsi of Landrace and Jinhua pigs (Jiang et al., 2019), revealing the potential biological role of m^6^A modification in regulating muscle growth and development.

#### 4.2.1 The critical role of m^6^A modulators in myogenic differentiation

A previous study found that after METTL3 knockdown in proliferating C2C12 cells, the overall levels of m^6^A modification decreased, resulting in premature differentiation of myoblasts, thus demonstrating that METTL3-mediated m^6^A methylation is an important regulator of myoblast state transition (Gheller et al., 2020). Both the mRNA and protein levels of METTL3/14 and WTAP were significantly downregulated during C2C12 cell differentiation and were negatively correlated with the expression of MHC and MEF2C. Consistent with *in vitro* findings, the expression of METTL3/14 is significantly downregulated in mouse embryonic hindlimb muscles during skeletal muscle growth and development (Xie et al., 2021a). Through genome-wide expression and gain/loss of function analysis, METTL3/14-mediated m^6^A methylation was found to inhibit myogenic differentiation by enhancing MNK2-ERK signaling (Xie et al., 2021a). This was consistent with the findings of Liang et al. (2021) and Gheller et al. (2020), suggesting that METTL3/14-mediated m^6^A methylation has a common inhibitory effect on myogenesis. However, METTL3 knockdown in proliferative C2C12 cells significantly downregulated MyoD mRNA expression and inhibited myoblast differentiation, and its m^6^A-mediated modification stabilized MyoD mRNA levels by promoting mRNA processing, thereby maintaining myoblast myogenic potential (Kudou et al., 2017). This is consistent with the findings of Zhang et al. (2020), who showed that METTL14 knockdown downregulates MyoD mRNA expression and significantly inhibits myotube formation.

Investigating METTL3/14 promotion or inhibition of myoblast differentiation, we found that m^6^A modification may mediate the expression of myogenic transcription factors by regulating specific signaling axes, thereby inducing myogenic differentiation. In contrast, the upstream of m^6^A-modified myogenic transcription factors may be regulated by other transcriptional elements in multiple ways, leading to the opposite result of myogenic differentiation. It has been suggested that m^6^A modification in myogenesis has multiple functions, but the current study only analyzed the potential mechanism of m^6^A modification-mediated differentiation induced by myogenic transcription factors. Future research needs to explore the key upstream/downstream factors co-regulated by RNA m^6^A methylation and myogenic transcription factors to explain the specific functional mechanism of m^6^A modification in regulating myogenic differentiation.

FTO-mediated m^6^A plays an important role in fat mass and lipogenesis (Song et al., 2020). Considering that the skeletal muscle participates in the body’s metabolic regulation and is similar to fat-related functions, it can be considered that FTO has an important regulatory effect on its myogenic differentiation. FTO expression is increased during the differentiation of mouse myoblasts into myotubes, whereas FTO silencing inhibits differentiation and impairs skeletal muscle development in endogenous FTO-null mice (Wang et al., 2017). Further exploration revealed that FTO-mediated m^6^A modification promoted myogenic differentiation through the mTOR-PGC-1α–mitochondrial axis. Interestingly, FTO overexpression, *in vitro*, does not significantly promote myoblast differentiation, presumably because of the high abundance of endogenous FTO expression, which is sufficient to support muscle differentiation (Wang et al., 2017). This is similar to the results of Church et al. (2010), who found that FTO overexpression did not increase lean mass in male mice.

Furthermore, the potential role of m^6^A modification in goat skeletal muscle was explored, and it was found that FTO-mediated m^6^A demethylation activity upregulates CCND1 expression in a YTHDF2-dependent manner and that silencing of FTO can also induce autophagy during myogenic differentiation (Deng et al., 2021b). Moreover, the global m^6^A modification in goat embryonic skeletal muscle was significantly higher than that in newborn fetuses. Functional studies have shown that FTO-mediated m^6^A modification can promote the expression of GADD45B and myogenic differentiation by activating the p38 MAPK pathway (Deng et al., 2021a).

These results provide a new insight that FTO promotes myogenesis by regulating the expression of related genes and may serve as a new target for myogenic differentiation.

#### 4.2.2 m^6^A methylation regulates skeletal muscle homeostasis and regeneration

MuSCs are required for maintaining skeletal muscle homeostasis and regeneration after injury. This study found that the proliferative activity of MuSCs was reduced after METTL3 knockdown, resulting in a significant decrease in both m^6^A modification and protein expression levels of key genes of the Notch signaling pathway. Furthermore, YTHDF1 is positively correlated with the mRNA translation efficiency of Notch signaling pathway components, revealing a novel post-transcriptional mechanism whereby the METTL3-m^6^A-YTHDF1 axis further controls MuSC fate and muscle regeneration by regulating the Notch signaling pathway (Liang et al., 2021). As a key factor in the MAPK signaling pathway, the protein kinase, MNK2, can target the phosphorylation-activated ERK signaling pathway and maintain muscle homeostasis (Hu et al., 2012; Maimon et al., 2014).

By establishing a mouse muscle injury regeneration model, it was found that METTL3/14-MNK2 could activate MuSCs and promote their proliferation in the early stages of muscle regeneration, thereby controlling ERK signaling (Xie et al., 2021a). These results suggest that the METTL3/14-m^6^A-MNK2-ERK signaling axis is required to regulate early muscle regeneration after acute injury. In the establishment of a BaCl_2_-induced mouse skeletal muscle injury model, the global m^6^A levels in muscle tissue were significantly increased 3 days after injury. In addition, m^6^A also plays an important regulatory function during the state transition of MuSCs (Gheller et al., 2020), indicating that m^6^A is a key epigenetic modifier of skeletal muscle regeneration.

#### 4.2.3 m^6^A methylation regulates skeletal muscle hypertrophic response

Maintaining skeletal muscle mass is critical for an organism’s health. Evaluation of the m^6^A modification signature of skeletal muscle hypertrophic growth in mice with mechanical overload revealed that global m^6^A levels and METTL3 expression were significantly increased in overloaded muscles (Petrosino et al., 2022). The myofiber-specific METTL3 mouse model was constructed using gain-of-function and loss-of-function experiments, and further validation revealed that m^6^A content and the myofiber cross-sectional area increased in muscles overexpressing METTL3. Moreover, METTL3 can regulate the post-transcriptional process of the myostatin pathway, and its mediated m^6^A methylation affects TGF-β superfamily signaling by inhibiting the translation of the activin receptor, Acvr2a mRNA, thereby promoting hypertrophic growth of the skeletal muscle (Petrosino et al., 2022). These results reveal a novel post-transcriptional mechanism to regulate muscle gene-specific expression, namely, that m^6^A modification regulates muscle growth through the translation of activin receptors and is required to maintain muscle mass and function *in vivo*.

#### 4.2.4 m^6^A may be an effective modulator for the treatment of muscle-related diseases

MuSC transition from quiescent to activated and proliferative to differentiated states after skeletal muscle injury and multiple m^6^A-modifying genes are associated with MuSC function during the differentiation process. When METTL3 is knocked down in primary murine MuSCs, the proliferation of MuSCs is slowed and the engraftment ability of their primary transplantation is enhanced, but their serial transplantation ability is lacking (Gheller et al., 2020).

In addition to FTO, ALKBH5 may also be involved in regulating mRNA processing and metabolism related to skeletal muscle growth and development. ALKBH5-mediated m^6^A modification plays an important role in FoxO3-dependent neurogenic muscle atrophy (Liu et al., 2022). To verify the specific mechanism, m^6^A-seq and Co-IP combined with loss-of-function experiments were performed, and it was found that ALKBH5 activates FoxO3 signaling in an m^6^A-HDAC4-dependent manner in denervated muscles, resulting in loss of skeletal muscle mass and denervation muscle atrophy (Liu et al., 2022). These results suggest that ALKBH5 may be a potential therapeutic target for the treatment of neurogenic muscle atrophy.

In conclusion, m^6^A is an essential epigenetic regulator of MuSC function; further work is needed to determine the fate of specific m^6^A-modified proteins based on their binding activity to muscle mRNA, which is expected to be better applied to treat muscle atrophy and other related diseases.

## 5 Transcriptional and post-transcriptional regulation of myogenesis

Transcriptional and post-transcriptional regulation at the RNA level can rapidly respond to the regulation of the corresponding mechanisms when biological functions occur. In eukaryotic cells, mRNA efficiently initiates protein synthesis by adding a 5′ 7-methyl-guanosine cap and a 3′ poly(A) tail. However, mRNA-directed protein synthesis is blocked by sequence-specific RNA-binding proteins (Sonenberg and Hinnebusch, 2009; Nwokoye et al., 2021). This way of promotion or repression reveals the importance of RNA transcriptional regulation. Early studies have found that one of the main functions of m^6^A in mammalian cells is to mediate mRNA degradation, suggesting a possible negative correlation between m^6^A methylation and mRNA stability and transcription levels (Wang et al., 2014a; Wang et al., 2014b; Liu et al., 2014; Ping et al., 2014). Like DNA methylation and histone modification, RNA methylation, an important post-transcriptional epigenetic modification, plays an important role in regulating gene expression in specific cell types (Heck and Wilusz, 2019).

Therefore, future studies are needed to elucidate the synergistic regulation of m^6^A and myogenic transcription factors to explain the cascade response of transcriptional and post-transcriptional regulation during myogenesis.

### 5.1 Interactions between m^6^A-related proteins and myogenic transcription factors

Studies have reported a critical role of MyoD methylation modification during myogenic differentiation. During C2C12 cell proliferation, high m^6^A levels in the 5′ UTR can promote the efficient processing of MyoD mRNA and actively maintain its stability. Furthermore, MyoD mRNA levels and myotube formation are significantly inhibited when METTL3 is knocked down (Kudou et al., 2017). Through the analysis of m^6^A at six prenatal stages in pigs, it was identified that the m^6^A reader, IGF2BP1, is continuously downregulated, which can regulate mRNA stability and translation (Huang et al., 2018). The same phenotypic changes were observed after knockdown of METTL14 and IGF2BP1 in C2C12 cells, inhibiting myoblast differentiation and significantly downregulating MyHC, MyoD, and MyoG expression (Zhang et al., 2020). These results suggest that the m^6^A modification-mediated key transcription factor, MyoD, is a positive regulator of skeletal muscle differentiation.

The Notch signaling pathway plays an important role in regulating MuSCs and muscle regeneration and is necessary for MuSC maintenance, activation, proliferation, and differentiation (Brack et al., 2008; Buas and Kadesch, 2010; Bjornson et al., 2012; Gerli et al., 2019). It was found that the mRNA molecules of the receptor (Notch2), transcription factor (RBPJ), and activator (MAML1) of the Notch signaling pathway in myoblasts are also regulated by m^6^A modification (Liang et al., 2021). Through *in vivo* and *in vitro* validation of m^6^A MeRIP-seq and cellular functions, it was found that METTL3-mediated m^6^A modification significantly inhibited the translation efficiency of Notch signaling pathway components, thereby promoting MuSC and muscle regeneration (Liang et al., 2021). These results revealed a novel post-transcriptional mechanism for m^6^A regulation of mRNA methylation of key transcription factors in MuSC fate and muscle regeneration.

MEF2C, a member of the myocyte enhancer factor 2 (MEF2) family, induces the expression of muscle-specific genes, mainly by binding to basic helix–loop–helix proteins in myogenic regulatory factors. It can also bind to the promoter and enhancer regions of transcription factors that assist in regulating myoblast differentiation during muscle development (Molkentin et al., 1995; Black and Olson, 1998; Kim et al., 2008). Recent studies have revealed that m^6^A levels of MEF2C mRNA are significantly increased during bovine myoblast differentiation, and its expression is post-transcriptionally regulated by m^6^A modifications. Through gain-of-function and loss-of-function analyses, METTL3 was found to regulate myogenic differentiation by promoting the translation of MEF2C mRNA in an m^6^A-YTHDF1-dependent manner (Yang et al., 2022a). Furthermore, it is worth noting that both the mRNA and protein levels of METTL3 were significantly increased in MEF2C-overexpressing myoblasts. Genomic analysis and ChIP-qPCR demonstrated that MEF2C binds directly to the METTL3 promoter as a transcription factor to promote its expression (Yang et al., 2022a). This positive feedback loop during myogenic differentiation explains the transcriptional and post-transcriptional cascade regulatory mechanisms.

Therefore, an in-depth study of the novel mechanisms of transcription factors in coordinating gene transcription and RNA m^6^A modification is required to shed more light on the functional mechanisms of skeletal muscle growth and development.

### 5.2 Mechanism of action of m^6^A-related proteins and non-coding RNAs during myogenesis

Myogenesis is a highly coordinated process involving multiple mechanisms, the programmed occurrence of which is controlled by specific genes and epigenetic modifications. An increasing number of studies have shown that non-coding RNAs (ncRNAs) or m^6^A-mediated transcriptional/post-transcriptional regulation play an important role in gene expression in skeletal muscle development. Moreover, m^6^A has been identified as a key regulatory factor that affects the function of ncRNAs, thus participating in body growth and development. Therefore, we have summarized recent studies to elucidate the molecular mechanisms of m^6^A-regulated miRNAs and long non-coding RNAs (lncRNAs) in myogenesis.

#### 5.2.1 Methyltransferase 3 regulates muscle-specific miRNAs through transcriptional and post-transcriptional regulation

miRNAs are a class of highly conserved small RNA molecules that participate in biological processes by degrading target genes or inhibiting post-transcriptional translation. During miRNA biogenesis, METTL3-mediated m^6^A methylation markers can promote the binding and processing of primary miRNA to DGCR8 and promote the maturation of miRNA in a global and non-cell-type-specific manner (Alarcon et al., 2015b). In addition, the m^6^A-labeled nuclear reader and effector, HNRNPA2B1, can interact with the DGCR8 protein to promote the processing of primary miRNAs (Alarcon et al., 2015a). These results are consistent with those of Alarcon et al. (2015b). This study found that METTL3 overexpression significantly downregulated the expression of muscle-specific miR-1a, miR-133a, miR-133b, and miR-206 in differentiated C2C12 cells and a mouse model of muscle injury regeneration. Combined with the immunoprecipitation results, it was demonstrated that METTL3 inhibited muscle-specific miRNAs through m^6^A modification of primary miRNAs during skeletal muscle differentiation (Diao et al., 2021). These results are contrary to the previous findings of Alarcon et al. (2015b).

Therefore, Diao et al. (2021) further explored the complex mechanism by which METTL3 inhibits muscle-specific miRNAs. They found that in differentiated C2C12 cells, METTL3 overexpression significantly suppressed the expression of myogenic transcription factors, MEF2A/C and SRF, whereas the expression of epigenetic regulators, HDAC1, HDAC4, and HDAC8, was significantly upregulated. The METTL3-overexpressing C2C12 cells were then subjected to MEF2C overexpression and HDAC inhibitor TSA treatment, and it was noted that the expression of miR-1a, miR-133a, miR-133b, and miR-206 was significantly upregulated (Diao et al., 2021). These results suggest that METTL3 represses muscle-specific miRNA expression by repressing MEF2C and promoting HDAC family epigenetic regulators. Taken together, METTL3 can repress muscle-specific miRNAs at the transcriptional and post-transcriptional levels and plays an important role in muscle function maintenance and anti-differentiation.

#### 5.2.2 The critical role of methyltransferase 3-mediated lncRNA in myogenesis

LncRNAs are mainly involved in regulating gene expression at the transcriptional and post-transcriptional levels. It has been reported that lncRNA can directly induce the binding of chromatin remodeling proteins to target genes and change histone modification or DNA methylation status according to specific genome sites, thus regulating the expression of functional genes (Chen and Xue, 2016). In addition to the aforementioned epigenetic modification, m^6^A-methylated lncRNAs have been well-characterized. For example, METTL3 binds to RBM15 and RBM15B in a WTAP-dependent manner and promotes lncRNA XIST-mediated gene silencing through m^6^A-YTHDC1 (Patil et al., 2016). Furthermore, lncRNAs are involved in a variety of biological processes, and their post-transcriptional regulation plays an essential role in myogenesis. Recent studies have found that during myogenesis, m^6^A methylation levels of lncRNA are positively correlated with the transcriptional abundance of lncRNA, and m^6^A methylation modifies lncRNA to positively or negatively regulate its adjacent mRNA. Functional verification showed that METTL3-mediated m^6^A-lncRNA Brip1os is involved in muscle differentiation by negatively regulating the expression of Tbx2 mRNA (Xie et al., 2021b), revealing a novel mechanism of post-transcriptional regulation during skeletal muscle development.

## 6 Summary and outlook

The discovery of RNA m^6^A modifications has greatly broadened our understanding of the mechanisms underlying gene expression regulation. In recent years, many studies have confirmed that m^6^A modifications are the important components of myogenic regulatory networks. Based on this, we summarized current research on m^6^A modifications in skeletal muscle growth and development and highlighted the mechanisms of transcriptional and post-transcriptional regulation of m^6^A modifications with myogenic transcription factors, which may help to better explore the synergistic regulation at different levels during myogenesis. In addition, other myogenic transcription factors are expected to be modified by m^6^A or regulate the expression of m^6^A-related proteins in the form of transcription factors, and the interaction of multiple factors uncovers new transcriptional regulatory mechanisms in myogenesis.

The state transition of MuSCs and myoblasts is accompanied by dramatic changes in transcriptional regulation. Transcriptional changes affecting the muscle state are influenced by various aspects of ncRNAs (miRNA, lncRNA, and circRNA), DNA methylation, histone modifications, and chromatin remodeling. The current study found that miRNAs, lncRNAs, and circRNAs have m^6^A modifications and that m^6^A exerts facilitative and inhibitory effects by altering the expression of targeted ncRNAs. However, it remains unclear if ncRNAs can participate in myogenic differentiation by regulating the stability of m^6^A-related enzymes, and the biological mechanisms by which the two crosstalk during skeletal muscle development are still unknown. Multiomics should be integrated to explore the interactions between m^6^A and other epigenetic modifications in skeletal muscle development and elucidate the functional signals, regulating skeletal muscle development.

The role of m^6^A-regulated RNA processing and metabolism in myogenesis requires further investigation. In summary, METTL3/14 was found to promote or inhibit myoblast differentiation, although the cause of this heterogeneity has not been identified. Therefore, future studies should exclude analytical errors due to tissue and cell heterogeneity, apply single-cell transcriptome technology to functional studies of myoblasts, and comprehensively explore the specific functional mechanisms of the synergistic regulation of RNA m^6^A methylation and myogenic transcription factors.

Moreover, we should focus on the special mechanism between m^6^A and functional cellular markers and look for “classic” drugs targeting new-type m^6^A-related enzymes to provide new targets and ideas for the early prevention of skeletal muscle aging and treatment of muscle metabolic diseases.
